# Evolutionary origin and functional specialization of Dormancy-Associated MADS box (DAM) proteins in perennial crops

**DOI:** 10.1186/s12870-022-03856-7

**Published:** 2022-10-05

**Authors:** Carles Quesada-Traver, Alba Lloret, Lorenzo Carretero-Paulet, María Luisa Badenes, Gabino Ríos

**Affiliations:** 1 Departamento de Citricultura y Producción Vegetal, Instituto Valenciano de Investigaciones Agrarias (IVIA), Carretera CV-315, Km 10.7, 46113 Moncada, Valencia Spain; 2 Department of Biology and Geology, University of Almería, Ctra. Sacramento s/n, 04120 Almería, Spain; 3 Centro de Investigación de Colecciones Científicas de la Universidad de Almería (CECOUAL), University of Almería, Ctra. Sacramento s/n, 04120 Almería, Spain

**Keywords:** SVP (gene family) evolution, Plant phenology, Bud development, Winter dormancy, Loquat

## Abstract

**Background:**

Bud dormancy is a phenological adaptation of temperate perennials that ensures survival under winter temperature conditions by ceasing growth and increasing cold hardiness. *SHORT VEGETATIVE PHASE* (*SVP*)-like factors, and particularly a subset of them named *DORMANCY-ASSOCIATED MADS-BOX* (*DAM*), are master regulators of bud dormancy in perennials, prominently Rosaceae crops widely adapted to varying environmental conditions.

**Results:**

SVP-like proteins from recently sequenced Rosaceae genomes were identified and characterized using sequence, phylogenetic and synteny analysis tools. SVP-like proteins clustered in three clades (SVP1–3), with known DAM proteins located within SVP2 clade, which also included *Arabidopsis* AGAMOUS-LIKE 24 (AthAGL24). A more detailed study on these protein sequences led to the identification of a 15-amino acid long motif specific to DAM proteins, which affected protein heteromerization properties by yeast two-hybrid system in peach PpeDAM6, and the unexpected finding of predicted DAM-like genes in loquat, an evergreen species lacking winter dormancy. *DAM* gene expression in loquat trees was studied by quantitative PCR, associating with inflorescence development and growth in varieties with contrasting flowering behaviour.

**Conclusions:**

Phylogenetic, synteny analyses and heterologous overexpression in the model plant *Arabidopsis thaliana* supported three major conclusions: 1) DAM proteins might have emerged from the SVP2 clade in the Amygdaloideae subfamily of Rosaceae; 2) a short DAM-specific motif affects protein heteromerization, with a likely effect on DAM transcriptional targets and other functional features, providing a sequence signature for the DAM group of dormancy factors; 3) in agreement with other recent studies, DAM associates with inflorescence development and growth, independently of the dormancy habit.

**Supplementary Information:**

The online version contains supplementary material available at 10.1186/s12870-022-03856-7.

## Background

The adaptation of perennial plants to seasonal variations in temperate climates relies on a tight adjustment of plant phenology to predictable environmental changes, with a drastic impact on plant fitness and survival. In fact, northern and southern boundaries of the geographical distribution of plant species appear to depend on fruit maturation date and chilling required for bud-break, respectively, two important milestones of plant phenology [1]. Environmental conditions, mostly temperature and light, play a key role in such phenological adjustment by entraining a barely known molecular calendar, which shares common genetic and regulatory features in plants and animals [2]. In temperate perennial plants, bud formation and winter dormancy ensure meristematic tissues (vegetative and reproductive) remain in a non-growing safer state, regardless of short temporary warmer periods, until a quantitative perception of environmental chilling fulfils the genetically-encoded chilling requirements of a given cultivar or variety [3]. This true dormancy (or endodormancy) term opposes to paradormancy and ecodormancy, which respectively refer to the quiescent state of buds repressed by correlative inhibition and dormancy-released buds requiring a period of warm temperatures prior to growth resumption and bud-break [4]. Since global warming is expected to reduce available winter chilling for satisfying winter dormancy release requirements, this climatic threat is potentially capable of impairing adaptability and yield in the case of crops [5], and even favouring the summer dormancy trait that enhances plant survival under extreme temperatures and summer droughts [6].

A set of MADS-box domain transcription factors related to *SHORT VEGETATIVE PHASE* (*SVP*) and *AGAMOUS-LIKE 24* (*AGL24*), involved in flowering time control in *Arabidopsis* [7, 8], have been reported to regulate bud formation and dormancy induction in evolutionarily distant perennials [9–12]. A particular group of these *SVP*-like genes named *DORMANCY-ASSOCIATED MADS-BOX* (*DAM*) has been found deleted in the *evergrowing* (*evg*) mutant of peach (*Prunus persica*) showing no growth cessation during winter [13]. Since then, numerous molecular and functional approaches have provided plenty of evidences about the implication of *DAM* genes in regulating the dormancy cycle in Rosaceae species [14, 15], namely in pear [16–19], apple [20–22], Japanese apricot [23–25], sweet cherry [26, 27] and peach [28, 29]. Also, *DAM* genes have been found transcriptionally associated with bud dormancy transitions in apricot [30], European plum [31], Chinese plum [32] and almond [33]. These studies depict a master role of *DAM* genes in promoting winter dormancy, modulated at the transcriptional level by seasonal cues, epigenetic modifications and plant hormones [14, 34].

Previous phylogenetic studies have identified three main clades within *SVP*-like group, most likely originated in an ancient whole-genome triplication [35]. In Rosaceae, two of these clusters have undergone independent lineage-specific gene expansion events due to whole-genome duplications [36] and tandem gene duplications in the Amygdaloideae subfamily (pears, apple, peach, plum and related crops), with potential effects on *SVP*-like and *DAM*-like functional diversification in bud dormancy and other processes [37]. In particular, *DAM* gene duplication and subsequent subfunctionalization events, leading to different seasonal expression patterns [38] and variable protein-interaction specificity resulting in alternative DNA-binding preferences, have been proposed to support the increased flexibility and complexity required for dormancy regulation in peach and apple [21, 39]. Here, we have scanned available genomes of Rosaceae species, most of them corresponding to crops with high agricultural and economical interest, to search for *SVP*-like and *DAM*-like genes and compared their encoded protein sequences in order to identify putative DAM-specific signatures as well as to explore their phylogenetic relationships. In addition, over these evolutionary approaches, we have reported the unexpected presence and expression of *DAM*-like genes in loquat (*Eriobotrya japonica*), an evergreen species lacking winter dormancy. Our results support the involvement of *DAM*-like genes in overall growth responses rather than a more restricted role in dormancy regulation, and provide a useful research model for the study of *DAM* functions deprived of winter dormancy determinants.

## Results

### Phylogenetic and synteny analysis supports the evolutionary origin and expansion of Amygdaloideae *DAM* genes within the *AthAGL24* containing *SVP2* clade

We built a comprehensive and well curated dataset of 87 S*VP* and S*VP-like* genes by scanning the genomes of ten Rosaceae perennial species with phenological and farming interest, including seven Amygdaloideae species, namely wild apple (*Malus baccata*), apple (*Malus x domestica*), Chinese pear (*Pyrus x bretschneideri*) and loquat (*Eriobotrya japonica*) from the Maleae tribe, and Japanese apricot (*Prunus mume*), peach (*Prunus persica*) and European plum (*Prunus domestica*) from the Amygdaleae tribe, as well as woodland strawberry (*Fragaria vesca*), black raspberry (*Rubus occidentalis*) and rose (*Rosa chinensis*) from the Rosoideae tribes Potentilleae, Roseae and Rubeae, respectively, plus five species from representative lineages of eudicots plants, namely jujube (*Ziziphus jujuba*), mulberry (*Morus notabilis*), *Arabidopsis thaliana*, kiwifruit (*Actinidia chinensis*), tomato (*Solanum lycopersicum*), plus the basal angiosperm *Amborella trichopoda* used as an outgroup (Table 1 and Supplementary Table S1). The list of the 87 detected SVP and SVP-like proteins with their corresponding genomic loci is shown in Supplementary Table S2.

**Table 1:** Number of *SVP*-like genes in the species under study

Family	Subfamily	Tribe	Species	SVP1	SVP2	SVP3	Total
Rosaceae	Amygdaloideae	Maleae	*Malus baccata*	2	5	0	7
			*Malus x domestica*	2	5	0	7
			*Pyrus x bretschneideri*	3	4	0	7
			*Eriobotrya japonica*	2	6	0	8
		Amygdaleae	*Prunus mume*	1	6	1	8
			*Prunus persica*	1	6	1	8
			*Prunus domestica*	1	6	1	8
	Rosoideae	Potentilleae	*Fragaria vesca*	1	5	0	6
		Roseae	*Rosa chinensis*	1	4	0	5
		Rubeae	*Rubus occidentalis*	1	2	1	4
Rhamnaceae			*Ziziphus jujuba*	1	1	1	3
Moraceae			*Morus notabilis*	1	1	2	4
Brassicaceae			*Arabidopsis thaliana*	1	1	0	2
Actinidiaceae			*Actinidia chinensis*	3	2	1	6
Solanaceae			*Solanum lycopersicum*	1	1	1	3
Amborellaceae			*Amborella trichopoda*	–	–	–	1

**Fig. 1:**
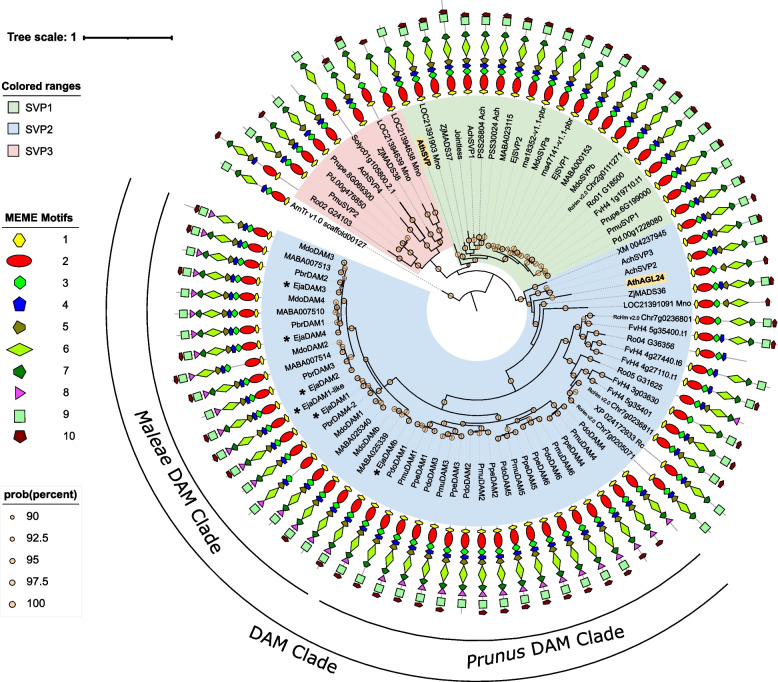
Maximum likelihood phylogenetic tree of SVP-like proteins from 10 Rosaceae species, five other eudicots and *Amborella trichopoda*. The tree was rooted using the single SVP-like sequence from the early diverging angiosperm *A. trichopoda* as an outgroup*.* The tree is drawn to scale, with branch lengths proportional to evolutionary distances between nodes. The scale bar indicates the estimated number of amino acid substitutions per site. Statistical support values on clades based on aLRT tests are represented as orange circles next to each node for those that resulted in posterior probabilities above 0.90, with diameters proportional to the actual value. The three main retrieved clades (SVP1, SVP2 and SVP3) are shown. Loquat SVP-like proteins are labelled with asterisks, and *Arabidopsis* AthSVP and AthAGL24 are highlighted. A schematic representation drawn to scale of conserved protein motifs detected using MEME is shown next to each sequence

Next, we examined the evolutionary relationships among the compiled set of sequences by constructing a ML phylogenetic tree (Fig. 1). The resulting tree topology returned three major groups of *SVP* genes previously described [35], named SVP1, SVP2 and SVP3, as independent clades (Fig. 1). SVP1 and SVP2 clades contained the *Arabidopsis* flowering genes *AthSVP* and *AthAGL24*, respectively, whereas no *Arabidopsis* representatives were found in SVP3 (Table 1). In general, Rosaceae species displayed a higher number of genes in the SVP2 clade (Table 1), particularly in species belonging to the Amygdaloideae subfamily with four to up to six genes in *Prunus* species located in neighbouring positions of their genomes (Supplementary Table S2), two to five genes in species belonging to the Rosoideae subfamily, and one in non-Rosaceae species, except for the Actinidiaceae kiwifruit, which showed two genes. Expansion in the SVP1 clade was restricted to species from the Maleae tribe, with 2–3 genes for one in the rest of species (Table 1), again except for the Actinidiaceae kiwifruit, which showed three genes (Table 1). In contrast, SVP3 representatives in Rosaceae showed a more scattered distribution, with only one gene found in each of the three *Prunus* species belonging to the Amygdaleae tribe, and none among Maleae and Rosoideae species, except for the Rubea black raspberry, which showed one gene. All together, these results suggest independent gene duplication and loss events would have promoted lineage-specific expansion and contraction within Rosaceae SVP1 and SVP2 clades.

The well-known bud dormancy regulatory DAM proteins from Amygdaloideae clustered within the SVP2 clade, as previously shown [35], leading to two robustly supported independent clades for species belonging to the Maleae and Amygdaleae tribes (Fig. 1). In addition to well-established DAM proteins from *Prunus*, *Malus* and *Pyrus*, six putative loquat proteins without prior molecular data clustered into the DAM clade, being thus plausible candidates to integrate the DAM family in this species.

**Fig. 2:**
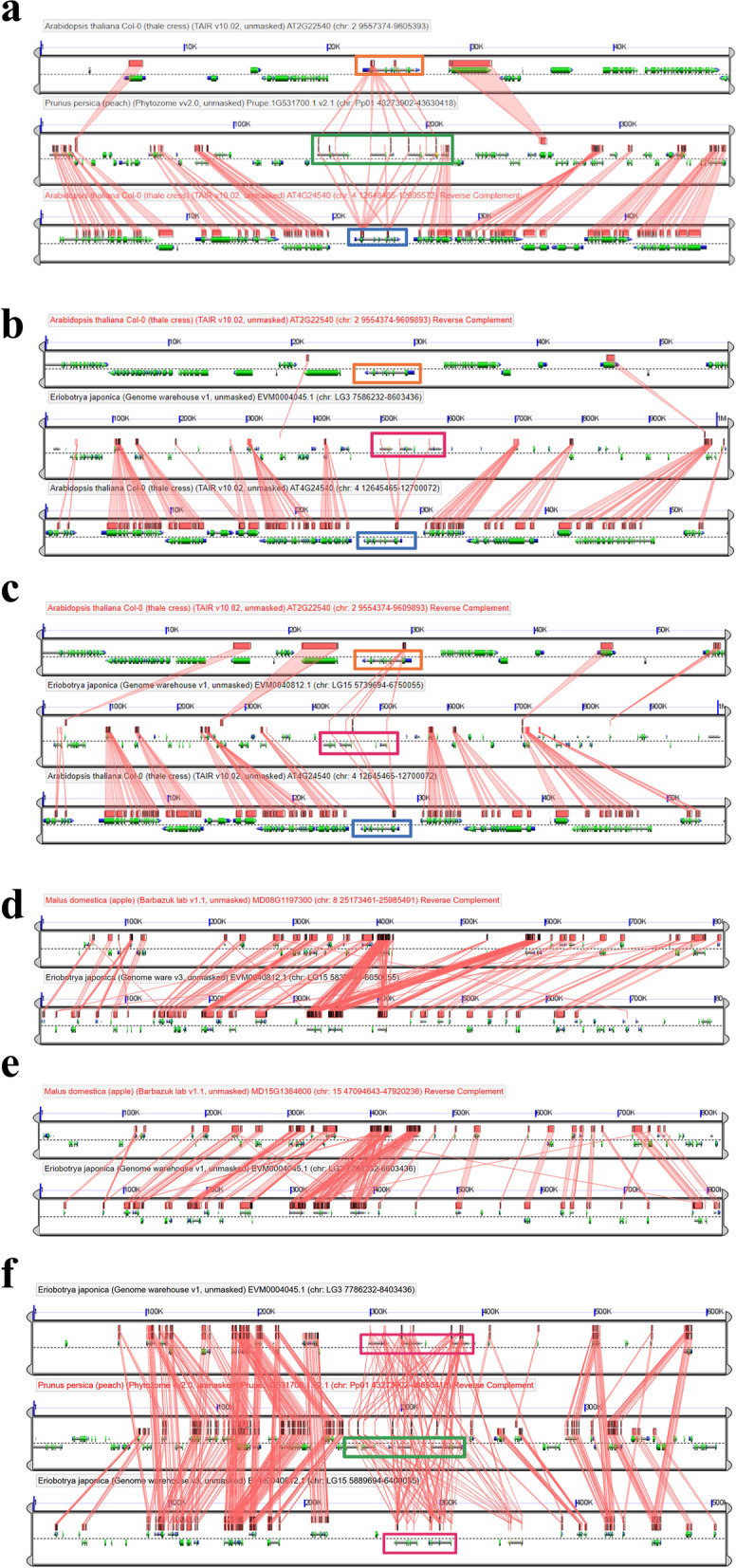
High resolution microsynteny analysis of genomic regions containing *SVP and SVP*-like genes from selected species. *Arabidopsis* and peach (**a**), *Arabidopsis* and loquat (**b**, **c**), apple and loquat (**d**, **e**), peach and loquat (**f**). In each pairwise comparison, putatively homologous sequences are connected by red edges. *Arabidopsis AthSVP* (orange box), *Arabidopsis AthAGL24* (blue box), peach *DAM* locus (green box) and loquat *DAM* loci (magenta box) are labelled

Phylogenetic analysis suggested the evolutionary origin of *DAM* genes prior to divergence of two main Amygdaloideae tribes during *SVP2* diversification. In order to seek additional evidence of the evolutionary relatedness between Amygdaloideae *DAM* genes and the *Arabidopsis AthAGL24* containing SVP2 clade, we performed in-depth microsynteny analysis among the genomic regions involved. The genomic region clustering the six tandemly-arrayed *DAM* genes from peach showed a higher number of putatively homologous genes collinearly arranged when compared to the *AthAGL24* genomic region than to the *AthSVP* one (Fig. 2a). Similarly, the two genomic regions, corresponding to linkage groups 3 and 5, which encompassed the six putative *DAM* genes from loquat identified in this study showed a strong signal of synteny when compared to *Arabidopsis AthAGL24* genomic regions, in contrast to what can be observed when compared to *AthSVP* ones (Fig. 2b,c). In addition, these two genomic regions in loquat had their respective syntenic counterparts in linkage groups 15 and 8 of the apple genome (Fig. 2d,e). A multiplex comparison of peach and loquat *DAM* loci supports the idea of lineage-specific Maleae DAM clades originating through a whole-genome duplications (WGD) event (Fig. 2f). In summary, i) microsynteny analysis further support the evolutionary origin of Amygdaloideae *DAM* genes from *AthAGL24* like genes in the SVP2 clade (Fig. 2), and ii) both WGD and tandem duplication events might have contributed to the expansion of the Amygdaloideae *DAM* gene family.

### *PpeDAM6* overexpression in *Arabidopsis* induces an *AGL24*-like phenotype

In order to get deeper insight into the origin of *DAM* genes, whether they are closer to *AthSVP* (clade SVP1) or *AthAGL24* (clade SVP2), we overexpressed a well characterized *DAM* gene in *Arabidopsis* and compared the phenotype of transformants with already published reports describing *AthSVP* and *AthAGL24* overexpressing lines. For easy availability and in-depth knowledge reasons, we chose the *PpeDAM6* gene from peach for that purpose.

**Fig. 3:**
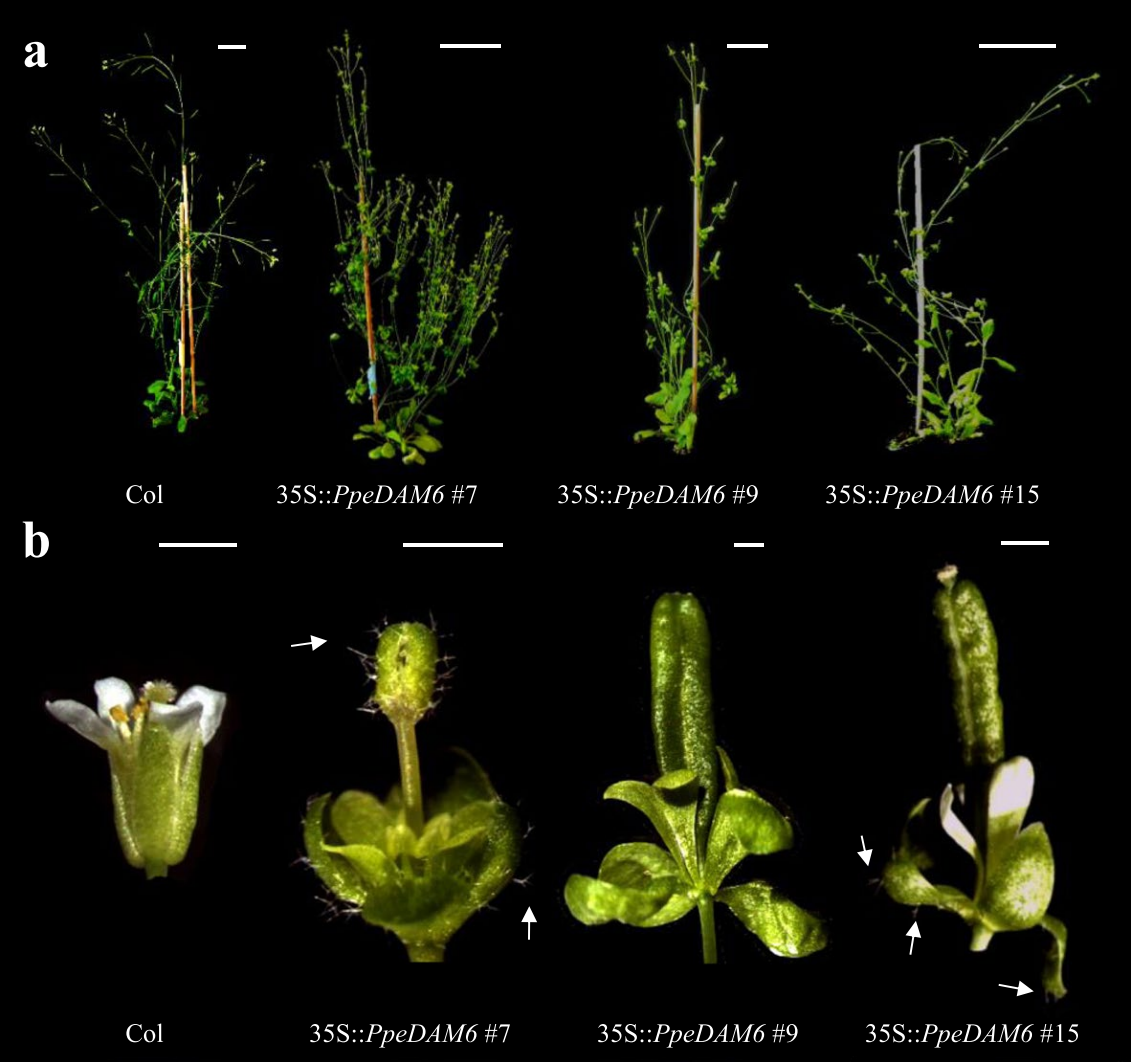
*PpeDAM6* overexpression affects flower development in *Arabidopsis*. Plant phenotype of wild-type Col (Columbia) and 35S::*PpeDAM6* lines #7, #9 and #15 (**a**). Flower alterations of these lines (**b**). Scale bars represent 5 cm (**a**) or 1 mm (**b**). A white arrow marks the presence of trichomes

The peach *PpeDAM6* gene was previously described at regulatory and functional levels [29, 40], being one of the best-known *DAM*-like genes. *PpeDAM6* was fused to c-myc epitope either in N-terminal or in C-terminal position and overexpressed under the control of the 35S promoter in *Arabidopsis*. At least 20 independent transgenic lines were obtained for each construction, showing qualitatively similar results. The transgenic lines displayed morphological abnormalities in floral structures at different degrees (Fig. 3), resembling floral defects of both the constitutive expression of *Arabidopsis AthSVP* [41] and *AthAGL24* [42]. The presence of the transgene and PpeDAM6 protein production was assessed by PCR and western-blot analysis. Although all the kanamycin-selected plants contained the transgene, PpeDAM6 protein accumulation was variable, in concordance with the severity of the observed phenotypic features (Supplementary Table S3). Accordingly, PpeDAM6 protein was not detected in most of transgenic plants showing wild-type phenotype, whereas plants with moderate protein expression showed mild defects and developed abnormal flowers with vegetative traits (trichomes) leading to defective siliques with no or few viable seeds. Some of them showed leafy sepals and normal petals (e.g. 35S::*PpeDAM6-*c-myc #15), and other lines had both leafy sepals and petals (e.g. 35S::c-myc-*PpeDAM6* #9) (Fig. 3b). On the other hand, plants expressing high levels of PpeDAM6 protein (e.g. 35S::*PpeDAM6-*c-myc #7) presented a more severe phenotype, with flowers replaced by inflorescences that often developed on the tip a new aberrant inflorescence without siliques (Fig. 3a,b). As noted above, most of these abnormal plants were sterile, with the exception of two lines showing few viable seeds.

We measured flowering time in genotype 35S::*PpeDAM6-*c-myc #15, which similarly to 35S::*AthAGL24* [8] and contrarily to 35S::*AthSVP* lines [7] showed early flowering phenotype (Table 2). This suggests that flowering time trait in *Arabidopsis* could be employed as a functional test for distinguish SVP1 and SVP2 clade proteins from even distant species, and that in close agreement with phylogenetic analyses *PpeDAM6* resembles SVP2 clade *AthAGL24* in functional overexpression studies.

**Table 2:** Flowering time of *Arabidopsis* transgenic lines with seeds. Flowering time was recorded as the rosette leaf number when the primary inflorescence stem appeared. An asterisk indicates significant difference compared with the control (Col) at a confidence level of 95%

Genotype	T1 Line	T2 Rossette leaf No. (*n* = 10)
Col		8.1 ± 1.2
35S::*PpeDAM6-c-myc#15*	1	6.9 ± 1.4*
	2	6.7 ± 1.0*
	3	7.9 ± 0.7
	4	6.8 ± 1.0*
	5	7.0 ± 1.3*
	6	6.8 ± 1.1*
	7	7.5 ± 1.5
	8	6.9 ± 1.1*
	9	7.2 ± 1.1*

### A characteristic MEME stretch of DAM proteins modifies protein interaction

**Fig. 4:**
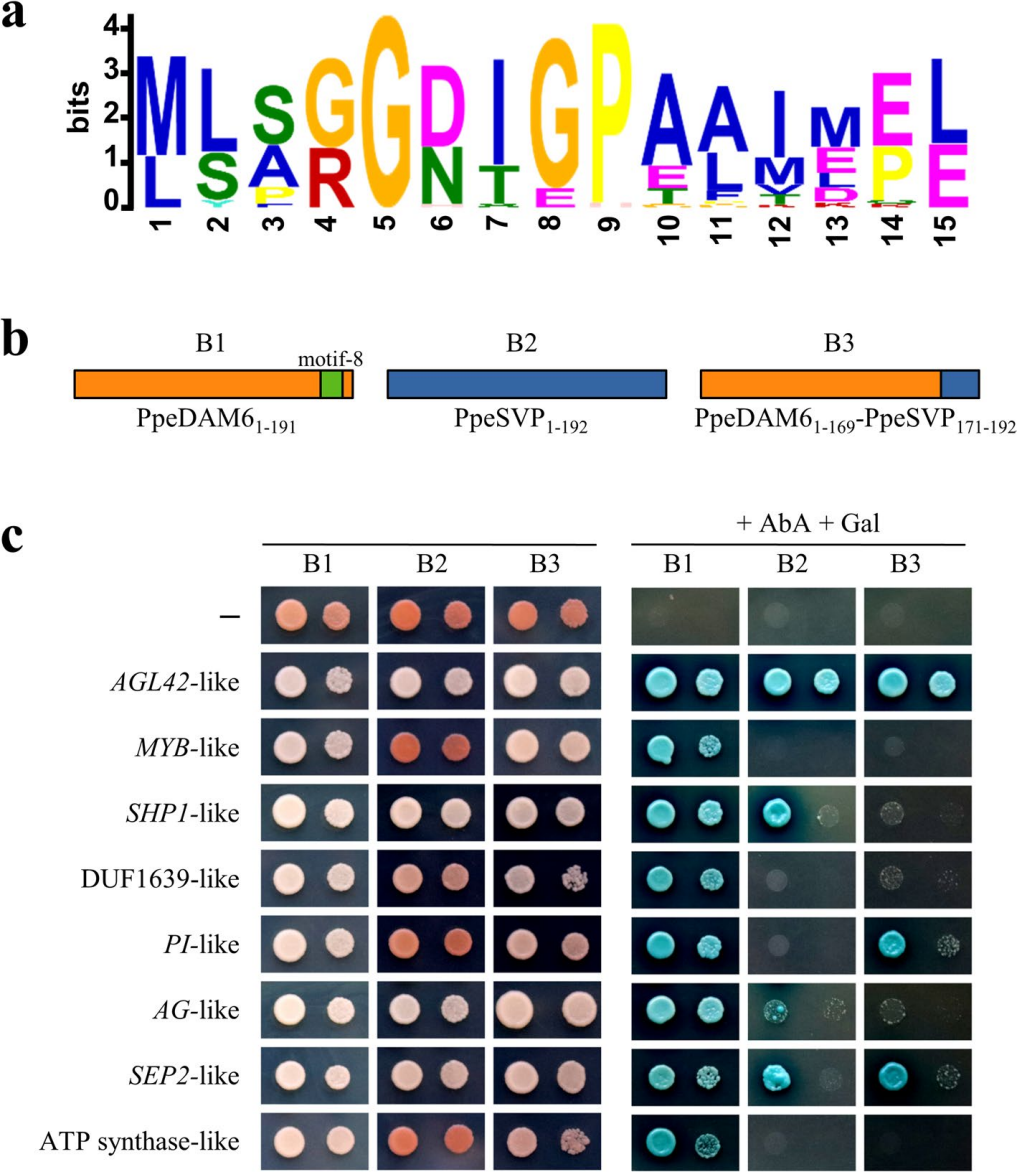
Replacement of a DAM-specific motif alters PpeDAM6 protein interactions. Logo representation of the 15 amino acid-long motif identified by MEME as conserved across DAM-like proteins (**a**). Bait constructs used in yeast two-hybrid (Y2H) experiments (**b**) including protein PpeDAM6_1–191_ with its natural DAM-specific motif in green (bait B1), PpeSVP_1–192_ (bait B2) and a chimerical construct of PpeDAM6 with the corresponding C-terminal end from PpeSVP (bait B3). Y2H analysis of protein interactions (**c**) between combinations of bait vectors (B1, B2 and B3) and prey vectors containing *AGL42*-like, *MYB*-like, *SHP1*-like, DUF1639-like, *PI*-like, *AG*-like, *SEP2*-like and ATP synthase-like genes. Yeast strains were grown on a minimal medium without tryptophan, leucine, histidine and adenine (left) and a chromogenic medium containing Aureobasidin A and X-α-Gal (+AbA +Gal) (right)

We used MEME for the identification of motifs conserved across our dataset of protein sequences (Supplementary Table S2). Setting the number of motifs to 10 allowed the identification of short sequences different from very well-known domains, such as MADS-box and K-box. A graphic display of MEME motifs distribution along proteins under study is shown in Fig. 1. A 15 amino-acid long motif (motif-8), located between the internal K-box domain and the C-terminal tail, was identified in 37 out of 38 sequences belonging to Amygdaloideae DAM clades (all but PmuDAM1) (Fig. 1), and in one additional SVP2-group protein from black raspberry that has not be described as a DAM member up to now (Ro05_G31625). A logo representation of this motif is shown in Fig. 4a. We next used this motif in FIMO searches against our set of protein sequences. According to FIMO, the whole group of 38 DAM proteins was positive for motif-8 occurrence with a q-value lower than 0.01, including PmuDAM1, and excluding Ro05_G31625 (Supplementary Table S4). Motif-8 could thus be considered as a DAM-specific motif in our set of genomes and be eventually used as a diagnostic motif in searches for *DAM* genes.

Since particular protein-protein interactions have been described to strongly condition the regulatory function of MADS-box transcription factors [43], we wondered whether this DAM-specific motif might contribute to modify the affinity of SVP-like factors for different protein partners, with a potential effect on the evolutionary development of the increased phenological plasticity observed in Rosaceae species. With that aim in view, we chose once more peach and *PpeDAM6* gene for a protein interaction assay with and without the DAM-specific motif. Then, we performed a yeast two-hybrid (Y2H) screening using peach *PpeDAM6* as bait in a construct lacking the C-terminal tail, due to its transcriptional auto-activating properties, on a peach bud-specific library [44]. The Y2H library contained 1.5 × 10^6^ independent clones, and 1.3 × 10^7^ interactions were tested, leading to 90 positive colonies. After positive rechecking and sequence analysis we obtained the protein interactors listed in Supplementary Table S5. A truncated clone of *PpeDAM6* encoding 191 amino acids of the protein, including the 15 residues-long DAM specific motif (bait B1, Fig. 4b), interacted with the MADS-box domain factors encoded by *AGAMOUS-LIKE 42* (*AGL42*)-like, *SHATTERPROOF 1* (*SHP1*)-like, *PISTILLATA* (*PI*)-like, *AGAMOUS* (*AG*)-like and *SEPALLATA 2* (*SEP2*)-like genes (Supplementary Table S5). Indeed, MADS-box factors are commonly forming heterocomplexes with other related MADS-box proteins [43]. We also identified a *MYB*-like gene, a gene encoding a domain of unknown function DUF1639, and a putative vacuolar ATPase gene (Supplementary Table S5).

In order to test the ability of the DAM-specific motif to alter the Y2H binding potential of these partners, we assayed two additional bait constructs: Prupe.6G199000 gene encoding peach SVP ortholog PpeSVP (bait B2), and a chimeric fusion of PpeDAM6 (amino acids 1–169) having the motif-8 sequence replaced by the collinear PpeSVP peptide (bait B3, Fig. 4b). The Y2H assay of AGL42-like, PI-like and SEP2-like was not drastically disturbed in B3 (Fig. 4c). However, the interaction of PpeDAM6 with MYB-like, SHP1-like, DUF1639-like, AG-like and ATPsynthase-like was severely reduced after motif-8 replacement, resembling PpeSVP behaviour. These results point to a modification of PpDAM6 structure as a result of motif-8 replacement, with impact on protein-protein interactions and presumably also protein functionality.

### *DAM*-like gene expression correlates with seasonal growth in loquat

**Fig. 5:**
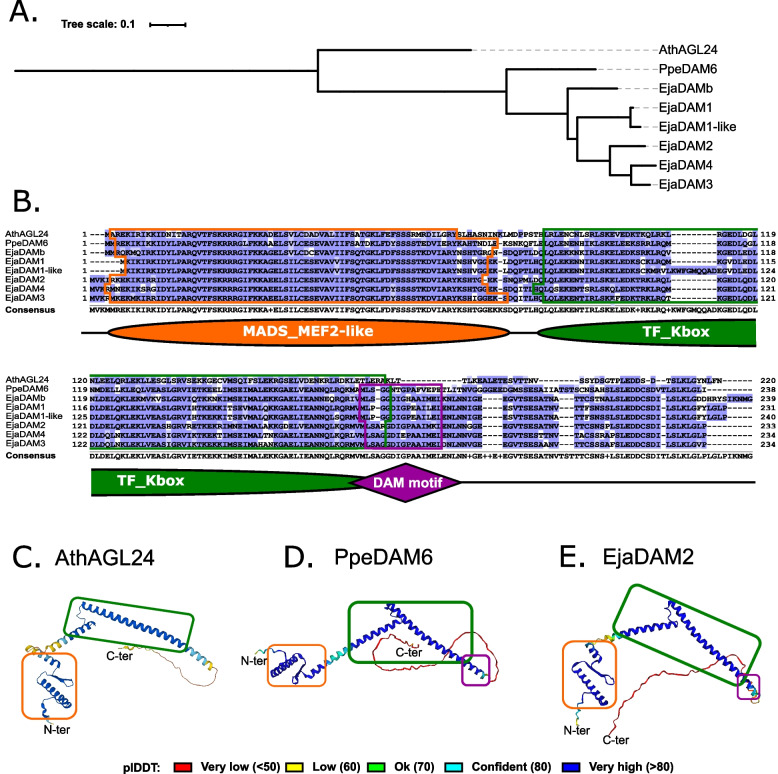
Protein sequence analysis of AthAGL24, PpeDAM6 and loquat DAMs. Pruned phylogenetic tree generated from the data presented in Fig. 1, only showing the proteins of interest (**a**). Multiple sequence alignment with residues coloured according to BLOSUM62 score. The consensus sequence and logo are shown below. InterPro predicted domains are noted in Orange and Green and the proposed DAM-specific motif is noted in purple (**b**). Protein structure prediction of AthAGL24 (**c**), PpeDAM6 (**d**) and EjaDAM2 (**e**) using ColabFold. Coloured boxes depict the domains and proteins are coloured according to plDDT that ranges from 0 to 100

We identified six novel *DAM*-like genes in loquat, which are homologs of previously described apple *DAM*s, in agreement with phylogenetic and syntenic studies (Figs. 1 and 2). Accordingly, these genes were named *EjaDAM1* (EVM0038001), *EjaDAM1*-like (EVM0001832), *EjaDAM2* (EVM0040812), *EjaDAM3* (EVM0017613), *EjaDAM4* (EVM0016705) and *EjaDAMb* (EVM0004045). To further investigate them, the tree shown in Fig. 1 was pruned including AthAGL24, PpeDAM6, and loquat DAMs (Fig. 5a). Multiple sequence analysis in combination with protein domain annotation evidenced clear similarities between loquat DAM proteins, AthAGL24 and PpeDAM6. All proteins exhibited a high degree of similarity, with AthAGL24 being the more distinct. Moreover, all harboured the MADS_MEF2-like and TF_Kbox domains, key components of MADS-box transcription factors (Fig. 5b). This similarity was also showcased by the structural predictions of AthAGL24, PpeDAM6 and EjaDAM2 (Fig. 5c-e).

**Fig. 6:**
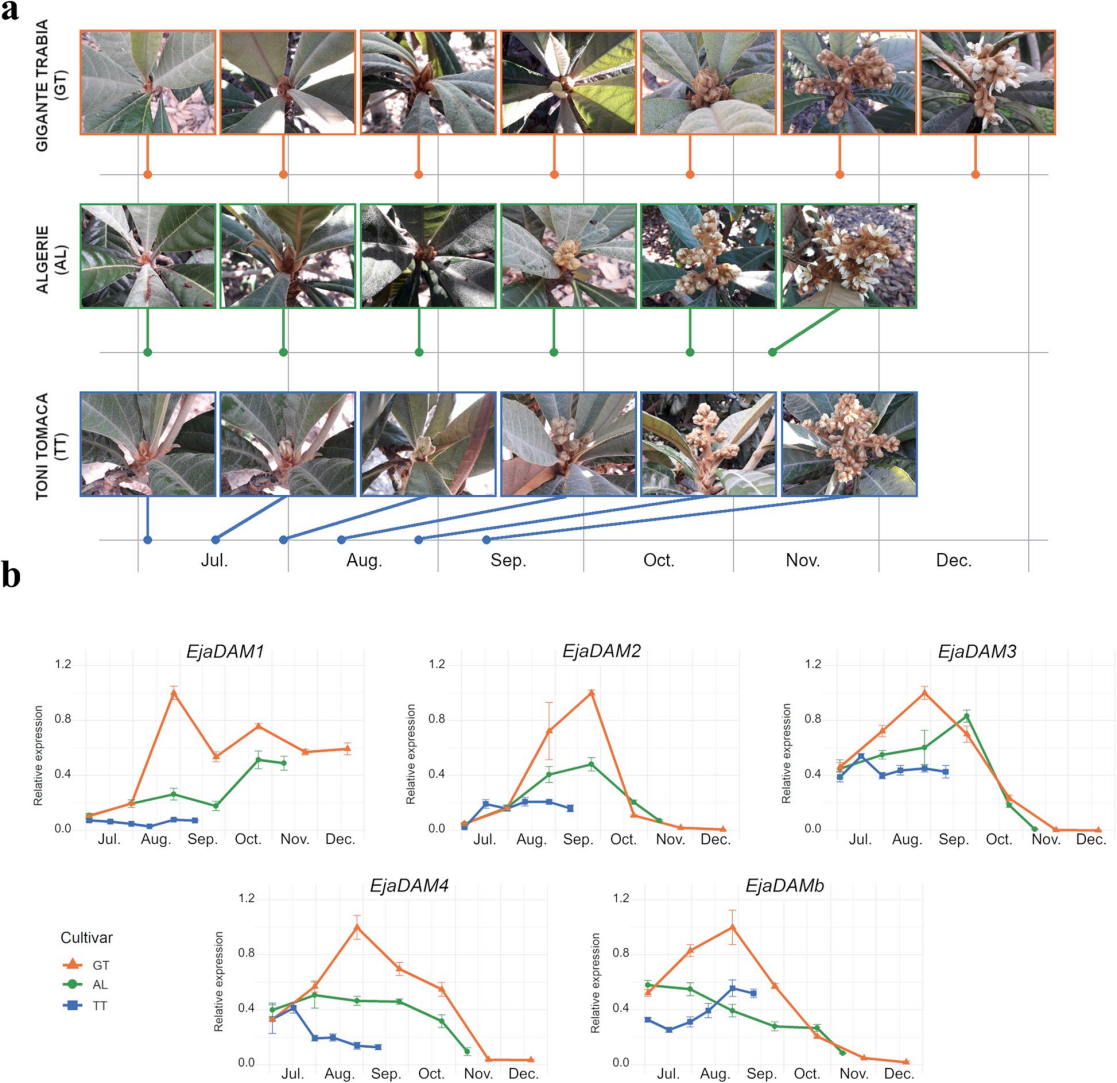
Flower development expression of *DAM*-like genes in loquat. Sampling of reproductive buds and inflorescences of three loquat varieties differing in flowering time: Gigante Trabia (GT, late), Algerie (AL, medium) and Toni Tomaca (TT, early) (**a**). Relative gene expression of *EjaDAM1–4* and *EjaDAMb* genes in these samples (**b**). An expression value of one is assigned to the highest sample. Data are means from three biological samples with two technical replicates each, with error bars representing standard deviation

Interestingly, loquat is an evergreen tree crop that flowers from the end of summer until winter, with large genotype-dependent differences. Consequently, loquat plants don’t show winter dormancy, although some authors have used the term summer dormancy to describe a seasonal period of growth rest at high temperatures [45]. Since *DAM* genes have been widely related to winter dormancy processes in temperate species, and *DAM* gene expression sharply decreases prior to bud growth resumption [14], we wondered if loquat *DAM*s were also developmentally regulated during flowering. We selected the varieties Toni Tomaca (TT), Algerie (AL) and Gigante Trabia (GT) as representatives of loquat genotypes with early, medium and late flowering habits, respectively (Fig. 6a). We collected inflorescence samples of them from the beginning of July until full blooming and analysed *DAM* gene expression by quantitative real time PCR (qRT-PCR). Overall, the expression level of the five *DAM* genes correlated with flowering dates, with higher peaks in the late GT and lower signals in the early TT, with gene-dependent particularities (Fig. 6b). *EjaDAM2*, *EjaDAM3*, *EjaDAM4* and *EjaDAMb* gene expression profile peaked in summer, prior to full inflorescence growth and development, and sharply decreased in the last two collected samples, with the exception of *EjaDAMb*, which specifically increased in full blooming samples of TT variety. On the other side, *EjaDAM1* expression increased in summer inflorescences and stayed stable during the duration of the experiment (Fig. 6b). These results are compatible with a wider role of *DAM* genes associated with growth repression, allowing flower development resumption under optimal environmental conditions, instead of a more restricted view of *DAM* as winter dormancy regulators.

## Discussion

### DAMs are phylogenetically and functionally related to the AGL24 subfamily of SVP proteins

Annual flowering and perennial dormancy processes are affected by environmental inputs by somehow analogous mechanisms [46–48]. Thus, it is not surprising to find the bud dormancy genes *DAM* within the SVP2 clade of MADS-box domain transcription factors including the *Arabidopsis* flowering regulator *AthAGL24. AthSVP* represses flowering by direct inhibition of the floral integrator *FT* [7, 49], whereas *AthAGL24* promotes flowering [8], and both *AthSVP* and *AthAGL24* determine floral meristem identity [50]. Consistently with these data, DAM proteins have been reported to bind the promoter of pear *FT2* gene during endodormancy [17]. In view of these precedents, the altered flowering development observed in transgenic *Arabidopsis* plants overexpressing *PpeDAM6* becomes easily understandable. Flower abnormalities observed in 35S::*PpeDAM6* plants strongly resemble the phenotype of *Arabidopsis* plants overexpressing *AthSVP* [41] and *AthAGL24* [8]. In addition, the overexpression in *Arabidopsis* of *PavDAM1–6* and *SVP*-like genes, which are involved in the dormancy process in *Prunus avium* and *Actinidia deliciosa* respectively*,* result in similar flower phenotypes defects [10, 27].

Interestingly, *AthSVP* and *AthAGL24* show opposite effects on flowering time in *Arabidopsis* by virtue of their different partners and targets [7, 8]. In this respect, the early flowering phenotype observed in a fertile 35S::*PpeDAM6* line in this study emplaces *PpeDAM6* closer to *AthAGL24* than to *AthSVP*. Such functional evidence corroborates phylogenetic and syntenic analyses arguing for a common origin of *AthAGL24* and *DAM* genes, both belonging to the SVP2 clade, in close agreement with previous phylogenetic studies of the SVP family [35, 37]. In line with these observations, the heterologous expression of apple *SVP* genes, but not the expression of apple *DAMs,* rescues the early flowering phenotype of the *Arabidopsis svp-41* mutant [21].

### Functional and structural specialization of DAM proteins in Rosaceae

Lineage-specific gene duplications events have been proposed to cause gene expansion and functional diversification in the *SVP* in Rosaceae [37]. Whereas SVP2 clade is expanded in the ten Rosaceae genomes included in this study, showing 2–6 members, SVP1 clade is specifically expanded in the four species of the Maleae tribe (Table 1). Such expansion and diversification of SVP-like factors has been proposed to support functional requirements for the perennial habit of growth of temperate climate trees, such as the formation of buds, the regulation of dormancy and the juvenile to mature transition [39]. The authors suggest the presence of subfunctionalization and/or neofunctionalization events in peach *DAM* genes [39], consistent with their different seasonal expression patterns [38]. In this respect, different heteromeric complexes of MdSVPa with MdDAM1, MdDAM4 and MdFLC resulted in different sets of transcriptional target genes, suggesting that these proteins performed non-redundant roles in dormancy [21]. Under this perspective, the lineage-specific expansion and subsequent functional diversification across Rosaceae genes belonging to the SVP1 and SVP2 clades might constitute the elemental basis of a finely tuned mechanism for a plastic genomic response under changing environments.

We have identified a short 15-residues motif specific to DAM and other related proteins from species belonging the Amygdaloideae subfamily including the genera *Malus*, *Pyrus*, *Eriobotrya* and *Prunus*, which formed a well-supported subclade within the SVP2 clade. This motif was found by the FIMO tool to be conserved in every DAM protein included in our dataset of examined sequences, and consequently can be considered as a DAM-specific signature, which can be tested on new species of Amygdaloideae as long as proteome annotations become available. Interestingly, the replacement of this DAM motif in PpeDAM6 by the collinear PpeSVP sequence affects its protein interaction capabilities (Fig. 4). Since the interaction of MADS-box domain proteins involved in dormancy in apple with different partners modifies their DNA-binding specificity and the set of downstream transcriptional targets [21], we may analogously infer that DAM-motif effect on PpeDAM6 interactions involves functional specialization with impact on gene regulatory networks.

### *DAM*s are not strictly linked to winter dormancy

Such previous phylogenetic and biochemical considerations contribute to shape a restricted DAM clade in Amygdaloideae subfamily (Fig. 1) with common structural and functional features, supposedly involved in bud dormancy regulation during winter time. This implies that certain *SVP*-like proteins from non-Amygdaloideae species, previously reported as DAM proteins, by virtue of their effect on bud growth and dormancy, could not strictly belong to this clade, such as DAM factors from leafy spurge [9], in spite of evident molecular and regulatory resemblances with DAMs in the clade [51]. On the other hand, loquat (and conceivably other related species with no available genomic data) shows consistent DAM proteins at the phylogenetic and molecular levels (Figs. 1, 6), but its physiological behaviour diverges from winter bud dormancy observed in perennial temperate plants belonging to the Amygdaloideae subfamily. Loquat is an evergreen tree that interrupts growth and flower development during the high temperatures of summer, and resumes normal growth and blooming in autumn and winter. Despite the fact that some authors describe this behaviour as summer bud dormancy [45], evidences supporting this rest as a true dormancy process, that is independent on environmental conditions until a given intrinsic regulated requirement is fulfilled, are lacking. Summer dormancy has been more extensively studied in herbaceous perennials than in woody ones, leading to the conclusion that phenological cycles of winter and summer dormant species are remarkably similar, but induced by symmetrical photoperiod and temperature environmental conditions [6]. Loquat could become an interesting woody model for comparatively studying the biochemical and molecular resemblances and differences between winter and summer dormancy adaptive strategies, but a deeper insight about its dormant behaviour is required beforehand. Regardless of such physiological details, *DAM* genes in loquat behave like genuine genotype-specific bud-growth repressor factors, characterized by a high gene expression in resting buds and also in cultivars with deeper or longer resting periods (Fig. 6). Such transcriptional patterns truly support the growing hypothesis that *DAM* genes are not strictly associated with winter dormancy events, but instead are more general growth regulatory factors impinging on meristematic cell division and hormonal balances, with impact on the growth-stress survival trade-off, as suggested by recent studies [25, 29].

## Conclusions

We provide an extensive compilation of SVP-like proteins deduced from recently sequenced genomes of the Rosaceae, a family of successful perennial plant species well adapted to temperate and tropical climatic conditions, which show a plastic and diverse response to winter temperatures by adjusting their bud dormancy requirements. Our data support a key role of *SVP*-like gene expansion and diversification, leading to the appearance of the *DAM* group in the subfamily Amygdaloideae within SVP2 clade, on this adaptive response. Amygdaloideae subfamily contains crops with a remarkable economic relevance such as apple, pear, peach, plum and almond, among others. We have identified a 15-amino acid long DAM-specific motif that constitutes a signature of known DAM factors. The absence of this motif impairs protein heteromerization with other regulatory factors, affecting thus to DAM transcriptional target specificity and function. We have analyzed *DAM*-like gene expression in the evergreen loquat, described as a summer dormancy species by some authors, to conclude that *DAM* expression associates with flower meristem activity and development in the important Amygdaloideae subfamily independently of the winter/summer dormancy habit.

## Methods

### Plant material

Loquat trees (*Eriobotrya japonica*) analysed in this study were grown at Instituto Valenciano de Investigaciones Agrarias, IVIA, Moncada, Spain, at 39° 34′ N, 0° 24′ W and 55 m above the sea level with drip irrigated silty-sandy soil at pH = 7,8. Flower buds from 3 varieties ranging in flowering time were harvested from the 3rd of July of 2020. The early blooming variety Toni Tomaca (TT) was harvested on 3/7, 17/7, 31/7, 12/8, 28/8 and 11/9. The intermediate blooming variety Algerie (AL) was harvested on 3/7, 31/7, 28/8, 25/9, 23/10 and 09/11. Finally, the latest blooming variety Gigante Trabia (GT) was harvested on 3/7, 31/7, 28/8, 25/9, 23/10, 23/11 and 21/12. Temperature data recorded during the timespan of the study is shown in Supplementary Fig. S1.

### Identification of SVP and SVP-like sequences

In order to obtain a reliable set of the *SVP* and *SVP-like* genes in representative plant species, their genomes (Supplementary Table S1) were scanned using well-characterized SVP-like protein sequences from *Arabidopsis thaliana*, namely AthSVP and AthAGL24, as queries in independent BLASTP searches. Sequences retrieved as best reciprocal hits [52] using one or another query were used to build a list of candidate gene (Supplementary Table S2). The list was completed using *SVP*-like and *DAM*-like genes previously reported in selected species and not included in our preliminary dataset [53], leading to a definitive list with their corresponding genomic loci shown in Supplementary Table S2. The retrieved sequences were further examined by means of sequence analysis tools [54] and their predicted gene models individually curated using GeneWise (https://www.ebi.ac.uk/Tools/psa/genewise/), with both their genomic DNA and protein sequences as input and settings left as default [55].

### Phylogenetic and protein motif analyses

Maximum Likelihood (ML) phylogenetic analysis was performed using PhyMLv3.1 [56] on the basis of multiple amino acid sequence alignments obtained using MUSCLE [57]. Prior to the analysis, the best fit amino acid substitution model by the AIC test was inferred using ProtTest v3.4.2 [58] to be JTT + G [59], i.e.*,* modelling heterogeneity in nucleotide substitution rates across positions in the alignment by means of a Gamma distribution with eight categories and an alpha shape parameter of 1.315. To optimize the search for the most likely tree topology, the best of NNI & SPR option (NNI, nearest-neighbor inter-change; SPR, subtree pruning and regrafting) was selected. Statistical significance on the retrieved topology was assessed by means of the Shimodaira–Hasegawa-like approximate likelihood ratio test [60].

Search for conserved motifs shared across protein sequences was performed with the Multiple Em for Motif Elicitation tool (MEME) suite v5.4.1 [61]., using the “zoops” site distribution (Zero or One Occurrence Per Sequence) set to 10 motifs and the rest of settings as default. Selected motifs resulting from MEME were used to scan the set of sequences using the Find Individual Motif Occurrences (FIMO) tool from the MEME suite [62]. Trees were edited and further annotated with the detected MEME protein motifs using interactive Tree Of Life (iTOL) v5 [63].

### Microsynteny analysis

Microsynteny analysis was conducted using the SynFind and Genome Evolution analysis (GEvo) tools from the Comparative Genomics platform (CoGe) [64]. First, syntenic regions between *Arabidopsis AthSVP* and *AthAGL24* and peach, apple and loquat genomes were searched using SynFind. The detected syntenic regions were further examined using GEvo. Non-coding regions were masked to include only protein-coding sequences and to ease visualization among comparisons. We used the default setting to define the minimum number of collinear genes for two regions to be called syntenic.

### *Arabidopsis* vectors and transformation

To overexpress *PpeDAM6* in *Arabidopsis*, a fragment containing full-length *PpeDAM6* fused to a N-terminal c-myc epitope was obtained from *PpeDAM6* cloned in pGBKT7 plasmid with specific primers (Supplementary Table S6) and then inserted into the pROK2 vector under the 35S promoter (35S::*c-myc-PpeDAM6*). A construct with the c-myc epitope fused to the C-terminal end of PpeDAM6 (35S::*PpeDAM6-c-myc*) was obtained using pGBKT7-*PpeDAM6* as a template in a PCR with specific primers (Supplementary Table S6). Both plasmids were introduced into *Agrobacterium tumefaciens* strain EHA105. *Arabidopsis* transformation was carried out by the floral dipping method [65]. Transformed seeds were selected on Murashige and Skoog (MS) medium supplemented with 50 μg/ml of kanamycin. Floral alterations were evaluated directly in T0 plants since many of them were sterile. For flowering time measurements, 10 T2 plants from 35S::*PpeDAM6* line #15 with abnormal phenotype but viable seeds were used. Seedlings were cultured in a chamber at 24 °C with a 16 h:8 h light-dark cycle.

### Western blot analysis

Protein extraction, western blot and immunological detection was performed according to [29]. Briefly, *Arabidopsis* leaves were boiled in Laemmli buffer during 10 min at 95 °C. Protein samples were resolved on sodium dodecyl sulphate-polyacrylamide gel electrophoresis (SDS-PAGE) on 15% resolving gel [66], and transferred to a polyvinylidene difluoride (PVDF) membrane (GE Healthcare-Life sciences). Membranes were incubated with Anti-myc Tag clone 4A6 (EMD Millipore) for 1.5 h, washed and then incubated for 1 h with anti-mouse IgG POD-secondary antibody (Roche). The BM chemiluminescence western blotting kit (Mouse/Rabbit) (Roche) was used for chemiluminescent detection.

### Yeast two-hybrid assay (Y2H)

A preliminary Y2H library screening using truncated *PpeDAM6* led to the isolation of several putative protein interactors [44]. The library construction and the Y2H screening was performed following Make Your Own “Mate & PlateTM” Library System and Matchmaker® Gold Yeast Two-Hybrid System (Clontech-Takara Bio) previously described in [67]. *PpeDAM6*, *PpeSVP* and a chimeric *PpeDAM6-PpeSVP* gene were cloned into pGBKT7 using primers shown in Supplementary Table S6, and introduced into yeast strain Y2HGold, using the Yeastmaker yeast Transformation System 2 (Takara Bio). Two-hybrid interactions were tested in minimal medium without tryptophan, leucine, histidine and adenine, and supplemented with Aureobasidin A (125 ng/ml) and X-α-Gal (125 μg/ml).

### Protein sequence analysis

Selected protein sequences (AthAGL24, PpeDAM6 and loquat DAMs) were subjected to Multiple Sequence Alignment (MSA) using ClustalW algorithm with default settings [54]. MSA results were processed for a proper interpretation using Jalview [68]. Protein structure prediction of the selected proteins was computed using ColabFold: a combination of MMseqs2 with AlphaFold2 [69].

### Analysis of gene expression by real-time quantitative PCR (RT-qPCR)

RNA extraction from loquat floral loquat buds was performed using a quick cetyltrimethylammonium bromide (CTAB) based procedure [70]. Potential contaminants were removed using RNase-Free DNase Set (Qiagen) following the manufacturer instruction. 500 ng of each sample were used for retrotranscription using the PrimeScript RT reagent kit (Takara Bio) in a total volume of 10 μL. RT-qPCR was conducted with 20x diluted samples on a StepOnePlus Real-Time PCR System (Life Technologies) using SYBR premix Ex Taq (Tli RNaseH Plus) (Takara Bio) with an initial incubation of 10 min at 95 °C, followed by 40 cycles of 15 s at 95 °C and 1 min at 60 °C each. Reaction specificity was tested by amplicon size estimation in electrophoresis and by melting curve analysis. For every datapoint three biological replicates with two technical replicates each were measured. *EjaActin* was used as the reference gene for all the experiments as previously described [53, 71]. The primers used in this study are listed in Supplementary Table S6.

## Supplementary Information

**Additional file 1: Fig. S1.** Environmental temperature variation corresponding to loquat sampling. **Table S1.** Plant genomes used in this study. **Table S2.** Proteins and genes shown in the phylogenetic tree. **Table S3.** Summary of *PpeDAM6* overexpressing *Arabidopsis* lines. **Table S4.** FIMO display of motif-8 occurrence in the set of SVP-like proteins. **Table S5.** Yeast 2-Hybrid (Y2H) interactors. **Table S6.** Primers used in this study.

## Data Availability

The datasets used and/or analysed during the current study were downloaded from the links and references listed in Table S1. Protein and DNA accession numbers are shown in Table S2, corresponding to the following databases and repositories: Genome Database for Rosaceae (GDR, https://www.rosaceae.org/), Genome Warehouse (GWH, http://bigd.big.ac.cn/gwh/), GenBank at the National Center for Biotechnology Information (NCBI, https://www.ncbi.nlm.nih.gov/), The Arabidopsis Information Resource (TAIR, https://www.arabidopsis.org/), Kiwifruit Genome Database (KGD, https://kiwifruitgenome.org/), Solanaceae Genomics Network (https://solgenomics.net/) and Phytozome (https://phytozome-next.jgi.doe.gov/).

## Abbreviations

AbA: Aureobasidin A
AG: Agamous
AGL24: Agamous-like 24
AL: Algerie
aLRT: Approximate likelihood-ratio test for branches
BLAST: Basic Local Alignment Search Tool
CoGe: Comparative Genomics platform
Col: Columbia
CTAB: Cetyltrimethylammonium bromide
DAM: Dormancy-Associated MADS-box
evg: Evergrowing
FIMO: Find Individual Motif Occurrences tool
FLC: Flowering Locus C
FT: Flowering Locus T
GEvo: Genome Evolution analysis tool
GT: Gigante Trabia
iTOL: Interactive Tree Of Life
MEME: Multiple Em for Motif Elicitation tool
ML: Maximum likelihood
MS: Murashige and Skoog medium
MSA: Multiple Sequence Alignment
MYB: Myeloblastosis
NNI: Nearest-neighbor inter-change
PI: Pistillata
PVDF: Polyvinylidene difluoride
qRT-PCR: Quantitative real time PCR
SDS-PAGE: Sodium dodecyl sulphate-polyacrylamide gel electrophoresis
SEP2: Sepallata 2
SHP: Shatterproof
SPR: Subtree pruning and regrafting
SVP: Short Vegetative Phase
TT: Toni Tomaca
WGD: whole-genome duplication
X-α-Gal: 5-Bromo-4-chloro-3-indoxyl-α-D-galactopyranoside
Y2H: Yeast two-hybrid
