# Supplementary material for: Evolutionary origin and functional specialization of Dormancy-Associated MADS box (DAM) proteins in perennial crops
Source: BMC Plant Biol. 2022 Oct 5;22:473. doi: 10.1186/s12870-022-03856-7 (PMC9533583; doi:10.1186/s12870-022-03856-7)
Supplement: Supplementary file 1 — Additional file 1: Fig. S1. Environmental temperature variation corresponding to loquat sampling. Table S1. Plant genomes used in this study. Table S2. Proteins and genes shown in the phylogenetic tree. Table S3. Summary of PpeDAM6 overexpressing Arabidopsis lines. Table S4. FIMO display of motif-8 occurrence in the set of SVP-like proteins. Table S5. Yeast 2-Hybrid (Y2H) interactors. Table S6. Primers used in this study. [file 12870_2022_3856_MOESM1_ESM.doc]

**Supplementary information**

**Fig. S1.** Environmental temperature variation corresponding to loquat sampling. Daily temperatures are shown (grey line). The temperature trend is shown (blue line) with confidence interval (grey shadow).


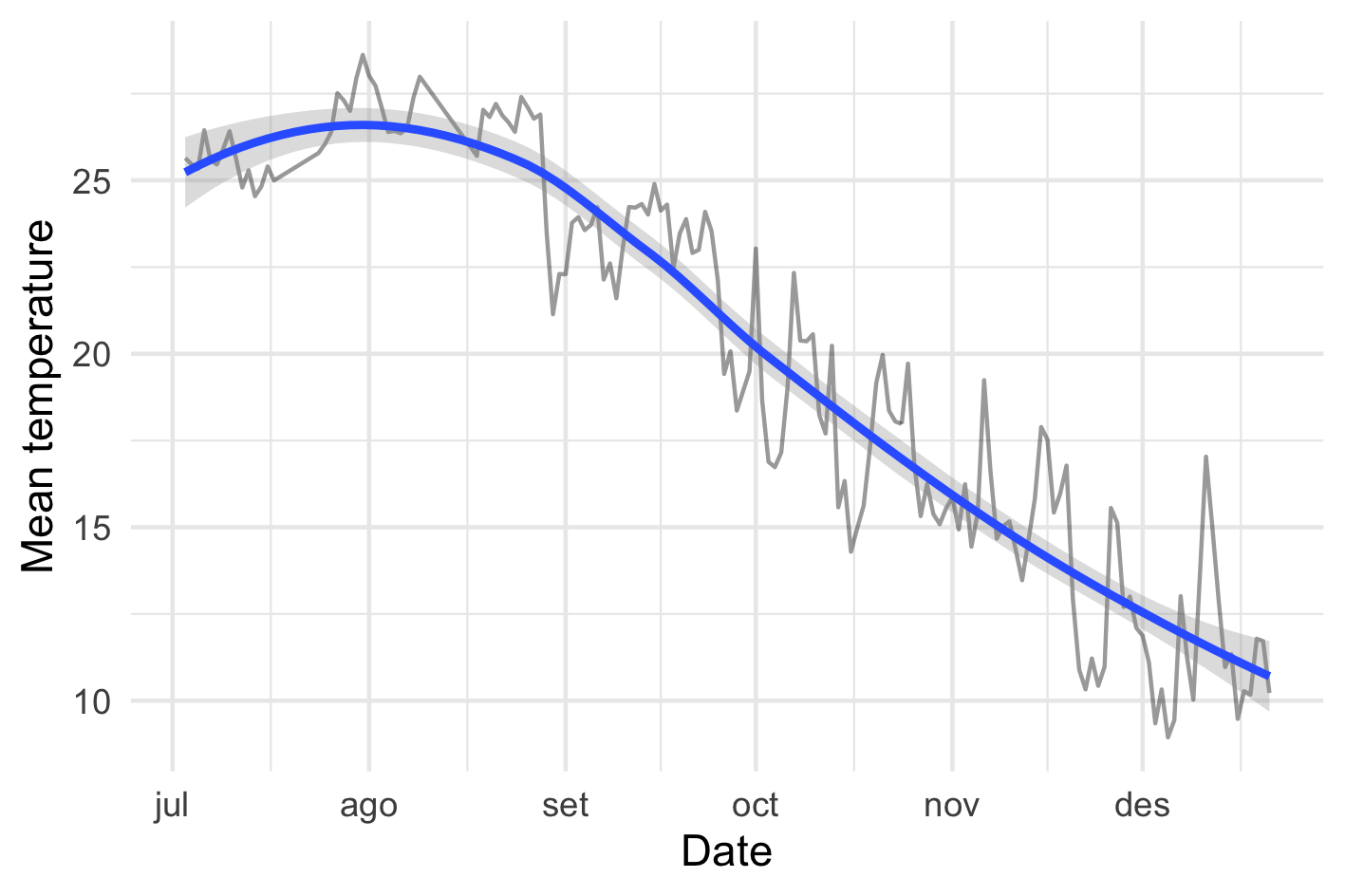


**Table S1.** Plant genomes used in this study.

| Species | Family | Order | URL | Reference |
| --- | --- | --- | --- | --- |
| *Malus baccata* | Rosaceae | Rosales | https://www.rosaceae.org/ | Chen *et al*. *G3 (Bethesda)* 2019; **9**: 2051–2060. |
| *Malus x domestica* | Rosaceae | Rosales | https://www.rosaceae.org/ | Daccord *et al.* *Nat Genet* 2017; **49**: 1099–1106 |
| *Pyrus x bretschneideri* | Rosaceae | Rosales | https://www.rosaceae.org/ | Xue *et al*. *BMC Genomics* 2018; **19**: 833 |
| *Eriobotrya japonica* | Rosaceae | Rosales | http://bigd.big.ac.cn/gwh/ | Jiang *et al*. *Gigascience* 2020; **9**: giaa015 |
| *Prunus mume* | Rosaceae | Rosales | https://www.ncbi.nlm.nih.gov/bioproject/171605 | Zhang *et al*. *Nat Commun* 2012; **3**: 1318 |
| *Prunus persica* | Rosaceae | Rosales | https://www.rosaceae.org/ | Verde *et al. BMC Genomics* 2017; **18**: 225 |
| *Prunus domestica* | Rosaceae | Rosales | https://www.rosaceae.org/ | Callahan *et al.* *Hortic Res* 2021; **8**: 8 |
| *Fragaria vesca* | Rosaceae | Rosales | https://www.rosaceae.org/ | Edger *et al*. *Gigascience* 2018; **7**: 1–7 |
| *Rosa chinensis* | Rosaceae | Rosales | https://www.rosaceae.org/ | Raymond *et al*. *Nat Genet* 2018; **50**: 772–777 |
| *Rubus occidentalis* | Rosaceae | Rosales | https://www.rosaceae.org/ | VanBuren *et al*. *Gigascience* 2018; **7** |
| *Ziziphus jujuba* | Rhamnaceae | Rosales | https://www.ncbi.nlm.nih.gov/bioproject/686508 | Huang *et al*. *PLoS Genet* 2016; **12**: e1006433 |
| *Morus notabilis* | Moraceae | Rosales | https://www.ncbi.nlm.nih.gov/bioproject/?term=PRJNA202089 | He *et al*. *Nat Commun* 2013; **4**: 2445 |
| *Arabidopsis thaliana* | Brassicaceae | Brassicales | https://www.arabidopsis.org/ | Cheng *et al*. *Plant J* 2017; **89**: 789–804 |
| *Actinidia chinensis* | Actinidiaceae | Ericales | https://kiwifruitgenome.org/ | Pilkington *et al*. *BMC Genomics* 2018; **19**: 257 |
| *Solanum lycopersicum* | Solanaceae | Solanales | https://solgenomics.net/ | Tomato Genome Consortium. *Nature* 2012; **485**: 635–641 |
| *Amborella trichopoda* | Amborellaceae | Amborellales | https://phytozome-next.jgi.doe.gov/info/Atrichopoda_v1_0 | Amborella Genome Project. *Science* 2013; **342**: 1241089 |

**Table S2**. Proteins and genes shown in the phylogenetic tree.

| Species | Name in the tree | Other name in databases | Genomic position |
| --- | --- | --- | --- |
| *Malus baccata* | MABA025340 | MABA025340 | scaffold487:271131..284230 |
|  | MABA007514 | MABA007514 | scaffold91:327118..341667 |
|  | MABA007513 | MABA007513 | scaffold91:287736..300961 |
|  | MABA007510 | MABA007510 | scaffold91:215527..227965 |
|  | MABA025339 | MABA025339 | scaffold487:240231..245540 |
|  | MABA023115 | MABA023115 | scaffold420:297365..307359 |
|  | MABA000153 | MABA000153 | scaffold1:3569716..3577142 |
| *Malus x domestica* | MdoDAM1 | NP_001315664 | NC_041803.1:47583719-47602775 |
|  | MdoDAM2 | NP_001315905 | NC_041796.1:25437125-25460450 |
|  | MdoDAM3 | XM_029107205 | NC_041796.1:25494224-25508030 |
|  | MdoDAM4 | XP_028963040 | NC_041796.1:25622968-25635046 |
|  | MdoDAMb | NP_001315741 | NC_041803.1:47617973-47647599 |
|  | MdoSVPa | NP_001280915 | NC_041789.1:13275746-13294044 |
|  | MdoSVPb | NP_001315811 | NC_041803.1:31506726-31516168 |
| *Pyrus x bretschneideri* | PbrDAM4-2 | XP_018500348 | NW_008988826.1-v1.1-pbr:23244..39649 |
|  | PbrDAM4-1 | XM_048583352 | NW_008988826.1-v1.1-pbr:10361..15455 |
|  | PbrDAM1 | XP_009376231 | chr8-v1.1-pbr:4395034..4408545 |
|  | PbrDAM2 | XP_009376230 | chr8-v1.1-pbr:4478973..4502558 |
|  | rna47141-v1.1-pbr | XP_009345716 | chr15-v1.1-pbr:23308199..23317632 |
|  | rna18352-v1.1-pbr | XP_009364259 | chr13-v1.1-pbr:9329214..9338213 |
|  | PbrDAM3 | LOC103964948 | chr8-v1.1-pbr:4518422..4532544 |
| *Eriobotrya japonica* | EjaDAM1 | EVM0038001 | LG3:8158196-8176579 |
|  | EjaDAM2 | EVM0040812 | LG15:6239694-6250644 |
|  | EjaDAM3 | EVM0017613 | LG15:6180869-6189642 |
|  | EjaDAM4 | EVM0016705 | LG15:6155422-6165225 |
|  | EjaDAMb | EVM0004045 | LG3:8085539-8103883 |
|  | EjaDAM1-like | EVM0001832 | LG3:8114185-8132912 |
|  | EjSVP1 | EVM0030394 | LG3:24416601-24421015 |
|  | EjSVP2 | EVM0025243 | LG16:20348255-20354409 |
| *Prunus mume* | PmuDAM1 | BAK78921 | NC_024127.1:4864482-4877401 |
|  | PmuDAM2 | BAK78922 | NC_024127.1:4851531-4867825 |
|  | PmuDAM3 | BAK78923 | NC_024127.1:4836374-4849866 |
|  | PmuDAM4 | BAK78924 | NC_024127.1:4827267-4839802 |
|  | PmuDAM5 | BAK78920 | NC_024127.1:4819663-4829577 |
|  | PmuDAM6 | BAH22477 | NC_024127.1:4808745-4821763 |
|  | PmuSVP1 | NP_001313437 | NC_024126.1:17171214-17179841 |
|  | PmuSVP2 | LOC103335885 | NC_024131.1:12980204-12991399 |
| *Prunus persica* | Prupe.8G069300 | Prupe.8G069300 | Pp08:10173458..10180572 |
|  | Prupe.6G199000 | Prupe.6G199000 | Pp06:20565244..20570006 |
|  | PpeDAM1 | XM_020563600 | NC_034009.1:43415242-43427535 |
|  | PpeDAM2 | PpeDAM2 | NC_034009.1:43424241-43463771 |
|  | PpeDAM3 | PpeDAM3 | NC_034009.1:43442855-43455102 |
|  | PpeDAM4 | PpeDAM4 | NC_034009.1:43452705-43464340 |
|  | PpeDAM5 | XM_020563613 | NC_034009.1:43461865-43474985 |
|  | PpeDAM6 | XM_007227500 | NC_034009.1:43471441-43482660 |
| *Prunus domestica* | PdoDAM1 | Pd.00g787580 | scaffold1404-v1.0:244863..251129 |
|  | PdoDAM2 | Pd.00g787570 | scaffold1404-v1.0:235501..243190 |
|  | PdoDAM3 | Pd.00g787550 | scaffold1404-v1.0:160860..170590 |
|  | PdoDAM4 | Pd.00g787540 | scaffold1404-v1.0:152061..159702 |
|  | PdoDAM5 | Pd.00g787530 | scaffold1404-v1.0:142761..151280 |
|  | PdoDAM6 | Pd.00g787520 | scaffold1404-v1.0:133574..142029 |
|  | Pd.00g1228080 | Pd.00g1228080 | scaffold2250-v1.0:346056..349662 |
|  | Pd.00g476850 | Pd.00g476850 | scaffold1428-v1.0:557962..563670 |
| *Fragaria vesca* | FvH4_1g19710.t1 | XP_011468575 | Fvb1_v4.0.a1:11921852..11930536 |
|  | FvH4_4g27110.t1 | XP_011464103 | Fvb4_v4.0.a1:28233227..28243434 |
|  | FvH4_4g27440.t6 | XP_011464115 | Fvb4_v4.0.a1:28434586..28444707 |
|  | FvH4_5g35400.t1 | XP_004299728 | Fvb5_v4.0.a1:25925249..25937305 |
|  | FvH4_5g35401 | XP_011464656 | Fvb5_v4.0.a1:25936753..25948338 |
|  | FvH4_3g03630 | XP_011460326 | Fvb3_v4.0.a1:2040554..2045222 |
| *Rosa chinensis* | RcHm_v2.0_Chr2g0111271 | LOC112190889 | RcHm_v2.0_Chr2:22880226..22889291 |
|  | RcHm_v2.0_Chr7g0236801 | LOC112180076 | RcHm_v2.0_Chr7:61513436..61535502 |
|  | RcHm_v2.0_Chr7g0236811 | LOC112180075 | RcHm_v2.0_Chr7:61531504..61544255 |
|  | RcHm_v2.0_Chr7g0205071 | LOC112178895 | RcHm_v2.0_Chr7:22723340..22741473 |
|  | XP_024172933 | LOC112178896 | NC_037094.1:22694569-22739342 |
| *Rubus occidentalis* | Ro01_G18500 | Ro01_G18500 | Ro01:10214281..10222796 |
|  | Ro02_G24103 | Ro02_G24103 | Ro02:27873301..27885143 |
|  | Ro05_G31625 | Ro05_G31625 | Ro05:37386600..37396535 |
|  | Ro04_G36356 | Ro04_G36356 | Ro04:8734093..8759269 |
| *Ziziphus jujuba* | ZjuMADS37 | XP_015890560.1 | NC_029686.1:12301151-12310153 |
|  | ZjuMADS36 | XP_015885971.1 | NC_029684.1:15883181-15893973 |
|  | ZjuMADS38 | XP_015893790 | NC_029687.1:24228013-24242585 |
| *Morus notabilis* | LOC21391903_Mno | LOC21391903 | NW_010365971.1:54662-66965 |
|  | LOC21394638_Mno | LOC21394638 | NW_010360493.1:62552-84385 |
|  | LOC21394639_Mno | LOC21394639 | NW_010360493.1:88881-123696 |
|  | LOC21391091_Mno | LOC21391091 | NW_010366098.1:32423-49904 |
| *Arabidopsis thaliana* | AthSVP | AT2G22540 | Chr2:9579874..9583893 |
|  | AthAGL24 | AT4G24540 | Chr4:12670965..12674072 |
| *Actinidia chinensis* | AchSVP1 | PSS21478 | LG9: 11,494,253-11,500,026 |
|  | AchSVP2 | PSS31583 | LG4: 5,554,137-5,560,621 |
|  | AchSVP3 | PSR99821 | LG21: 4,101,565-4,114,681 |
|  | AchSVP4 | PSS02789 | LG19: 10,206,029-10,214,193 |
|  | PSS30024 | PSS30024 | LG5: 2,336,122-2,348,891 |
|  | PSS26804 | PSS26804 | LG7: 13,516,997-13,523,715 |
| *Solanum lycopersicum* | Jointless | Solyc11g010570.2.1 | SL2.50ch11:3640857..3645766 |
|  | XM_004237945 | Solyc04g076280.2.1 | SL2.50ch04:61203256..61209308 |
|  | Solyc01g105800.2.1 | Solyc01g105800.2.1 | SL2.50ch01:93839471..93848297 |
| *Amborella trichopoda* | scaffold00127.17 | scaffold00127.17 | AmTr_v1.0_scaffold00127:599439..654192 reverse |

**Table S3.** Summary of *PpeDAM6* overexpressing *Arabidopsis* lines indicating their phenotype features, the presence of seeds, the detection of the transgen by PCR and protein level by western-blot.

| Genotype | Line | Phenotype features | Seeds | PCR | Western |
| --- | --- | --- | --- | --- | --- |
| Col |  | WT | Yes | - | nd |
|  |  | WT | Yes | - | nd |
|  |  | WT | Yes | - | nd |
| 35S::*c-myc-PpeDAM6* | 1 | WT | Yes | + | nd |
|  | 2 | WT | Yes | + | nd |
|  | 3 | WT | Yes | + | nd |
|  | 4 | WT, some leafy sepals | Yes | + | nd |
|  | 5 | WT | Yes | + | + |
|  | 6 | Leafy sepals, leafy petals, no siliques | Sterile | + | ++ |
|  | 7 | Leafy sepals, abnormal siliques | Sterile | + | ++ |
|  | 8 | WT | Yes | + | nd |
|  | 9 | Leafy sepals, leafy petals, abnormal siliques | Sterile | + | + |
|  | 10 | Leafy sepals, leafy petals, abnormal siliques | Sterile | + | ++ |
|  | 11 | Leafy sepals, leafy petals, no siliques | Sterile | + | +++ |
|  | 12 | WT | Yes | + | nd |
|  | 13 | WT | Yes | + | nd |
|  | 14 | Leafy sepals, abnormal siliques | Few | + | nd |
|  | 15 | Leafy sepals, leafy petals, no siliques | Sterile | + | +++ |
|  | 16 | Leafy sepals, leafy petals, abnormal siliques | Sterile | + | ++ |
|  | 17 | WT | Yes | + | nd |
| 35S::*PpeDAM6-c-myc* | 1 | WT | Yes | + | nd |
|  | 2 | WT | Yes | + | nd |
|  | 3 | WT | Yes | + | nd |
|  | 4 | WT, some leafy sepals | Yes | + | nd |
|  | 5 | WT | Yes | + | nd |
|  | 6 | Leafy sepals, leafy petals, no siliques | Sterile | + | ++ |
|  | 7 | Leafy sepals, leafy petals, no siliques | Sterile | + | ++ |
|  | 8 | Leafy sepals, leafy petals, abnormal siliques | Sterile | + | ++ |
|  | 9 | WT | Yes | + | nd |
|  | 10 | WT | Sterile | + | nd |
|  | 11 | WT, some leafy sepals | Yes | + | nd |
|  | 12 | WT | Yes | + | nd |
|  | 13 | WT | Yes | + | nd |
|  | 14 | WT | Yes | + | nd |
|  | 15 | Leafy sepals, abnormal siliques | Few | + | + |
|  | 16 | WT, some leafy sepals, abnormal siliques | Yes | + | ++ |
|  | 17 | WT | Yes | + | nd |
|  | 18 | WT | Yes | + | nd |

(+) indicated presence of transgen in PCR column and signal intensity in western column (more (+) indicated stronger signal); nd, not detected.

**Table S4.** FIMO display of motif-8 occurrence in the set of SVP-like proteins.

***
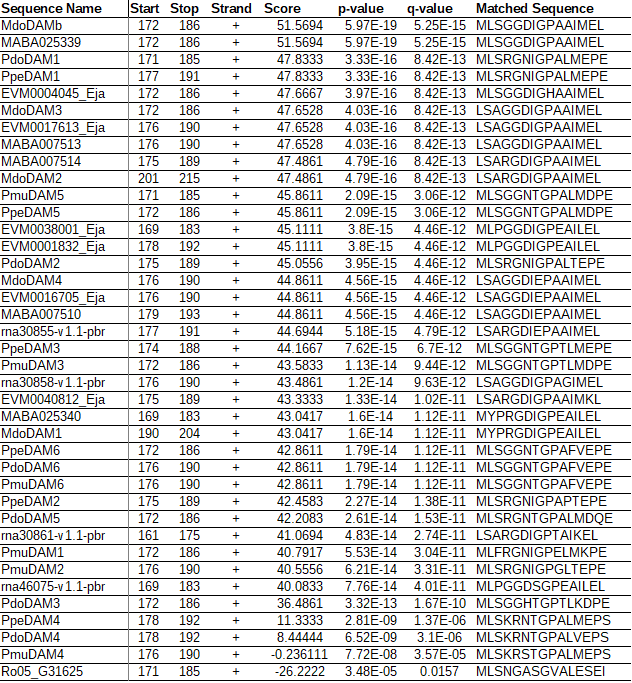
***

**Table S5.** Yeast 2-Hybrid (Y2H) interactors.

| Y2H positive | Peach transcript | *Arabidopsis* homolog | Functional domain |
| --- | --- | --- | --- |
| AGL42-like | Prupe.2G287500 | AT5G62165 (*AGL42*) | MADS-box transcription factor |
| MYB-like | Prupe.1G405400 | AT2G16720 (*MYB7*) | SANT/Myb domain |
| SHP1-like | Prupe.3G170600 | AT3G58780 (*SHP1*) | MADS-box transcription factor |
| DUF1639-like | Prupe.1G415100 | AT1G55340 | DUF1639 |
| PI-like | Prupe.1G489400 | AT5G20240 (*PI*) | MADS-box transcription factor |
| AG-like | Prupe.4G070500 | AT4G18960 (*AG*) | MADS-box transcription factor |
| SEP2-like | Prupe.3G249400 | AT3G02310 (*SEP2*) | MADS-box transcription factor |
| ATP synthase-like | Prupe.6G296700 | AT4G11150 | Vacuolar H^+^-ATPase |

**Table S6.** Primers used in this study.

|  | **Primers forward and reverse** |
| --- | --- |
| **pROK2 cloning** |  |
| *PpeDAM6-c-myc* | GCGTCTAGATGATGAGGGAGAAGATCAAGATCAAG  TATGGATCCTACAGGTCCTCCTCTGAGATCAGCTTCTGCTCGGGAAGCCCCAGTTTGAG |
| *c-myc-PpeDAM6* | CAGTCTAGATGGAGGAGCAGAAGCTGATCTC  AAAGGATCCCTAGGGAAGCCCCAGTTT |
| PCR test on genomic DNA | TACTGGACCTGCGTTTGTGGAGCC  TGTTGCAGCTGGTGGAGGTGGCAATT |
| **Two-hybrid cloning** |  |
| PpeDAM6 | ACCGAATTCATGATGAGGGAGAAGATCAAG  AAAGGATCCCTAGGGAAGCCCCAGTTT |
| PpeDAM6_1-191_ | AGGGAATTCATGATGAGGGAGAAGATCAAG  ATTGGATCCTTAATTAGTAATCAACGTCTCCGG |
| PpeSVP_1-192_ | ACAGAATTCATGGCGAGGGAGAAGATTC  ATTGGATCCTTACTCCTCCATAACTATGTTTTCTG |
| PpeDAM6-PpeSVP | AGGGAATTCATGATGAGGGAGAAGATCAAG  CTCCAGCACCTTCTGCCTTAACTGGTTGTT  AAGGCAGAAGGTGCTGGAGAAACATAATAGC  ATTGGATCCTTACTCCTCCATAACTATGTTTTCTG |
| **qRT-PCR** |  |
| *EjaDAM1* | GATAGAAGCTAACAACCAGCTAC  TCCAACTCCAGGATGGCCTCT |
| *EjaDAM1-like* | AGAGCTGCAAGCTGAGAGTGC  CAGCTCAGCTCCCTTTTTCTG |
| *EjaDAM2* | GGGGAAAAATCGAATCAACCGAT  CATAATCCTTTTTTCCTTAGTTTCCC |
| *EjaDAM3* | GTGAGATTATGGCACATGCGAAT  TTCGAGAGAAAAGGCACTGCTA |
| *EjaDAM4* | GTGAGATTATGGCACTTACGAAC  GTCATCTTCGAGAGAAGGAGCT |
| *EjaDAMb* | GCTGATAGAAGCTAATAACGAGCA  TCATCTTCAAGAGAAAGAGCATTTG |
| *EjaActin* | GGATTTGCTGGTGATGATGC  CCGTGCTCAATGGGATACTT |
